# Three-dimensional nanostructure determination from a large diffraction data set recorded using scanning electron nanodiffraction

**DOI:** 10.1107/S205225251600943X

**Published:** 2016-07-04

**Authors:** Yifei Meng, Jian-Min Zuo

**Affiliations:** a Department of Materials Science and Engineering, University of Illinois at Urbana-Champaign, 1304 W. Green Street, Urbana, IL 61801, USA; b Fredrick Seitz Materials Research Laboratory, University of Illinois at Urbana-Champaign, 104 S. Goodwin Avenue, Urbana, IL 61801, USA

**Keywords:** three-dimensional nanostructure, scanning electron diffraction, diffraction tomography, inorganic materials, nanocrystalline TiN films, crystal morphology

## Abstract

A diffraction-based technique is developed for the determination of three-dimensional nanostructures. The technique employs high-resolution and low-dose scanning electron nanodiffraction (SEND) to acquire three-dimensional diffraction patterns, with the help of a special sample holder for large-angle rotation. Grains are identified in three-dimensional space based on crystal orientation and on reconstructed dark-field images from the recorded diffraction patterns. Application to a nanocrystalline TiN thin film shows that the three-dimensional morphology of columnar TiN grains of tens of nanometres in diameter can be reconstructed using an algebraic iterative algorithm under specified prior conditions, together with their crystallographic orientations. The principles can be extended to multiphase nanocrystalline materials as well. Thus, the tomographic SEND technique provides an effective and adaptive way of determining three-dimensional nanostructures.

## Abstract 2

A general approach is developed to determine three-dimensional grain morphology and orientation in single-phase and nanocrystalline materials.

## 1. Introduction   

Nanocrystalline materials in general feature a high density of grain boundaries and a large surface-to-volume ratio, and they have attracted tremendous interest for their unique mechanical, chemical and electronic properties. For example, nanocrystalline metals or alloys exhibit improved hardness (Meyersm & Ashworth, 1982 ▸), enhanced strength (Abe *et al.*, 2002 ▸) and reduced ductility (Koch *et al.*, 1999 ▸), and the development of battery materials has benefitted greatly from nanostructured materials for improved capacity (Wang *et al.*, 2009 ▸; Brezesinski *et al.*, 2010 ▸), conductivity and mechanical stability (Gao *et al.*, 2013 ▸). The structure of a nanocrystalline material is determined by the constituent phases, composition, three-dimensional grain morphology, orientation and distribution, details of which can only be obtained from a three-dimensional structure determination, which is an outstanding challenge in crystallography (Billinge & Levin, 2007 ▸).

Previously, three-dimensional X-ray diffraction microscopy (3D-XRDM; Chapman *et al.*, 2006 ▸) was developed for the study of polycrystalline materials. Recently two new X-ray diffraction (XRD) techniques, namely differential-aperture X-ray microscopy (DAXM; Larson *et al.*, 2002 ▸) and diffraction contrast tomography (DCT; Ludwig *et al.*, 2008 ▸), achieved a submicron spatial resolution in three dimensions. Using a combination of a scanning electron microscope (SEM) and a focused ion beam, three-dimensional electron backscattered diffraction (3D-EBSD; Rollett *et al.*, 2007 ▸) is the technique for obtaining three-dimensional orientation maps in bulk polycrystalline samples. However, the spatial resolution in an SEM is limited to tens of nanometres. Also, the destructive nature of 3D-EBSD makes it unfavourable for multi-technique or *in situ* analysis.

Various transmission electron microscope (TEM)-based techniques have been established for the two-dimensional orientation mapping of nanocrystalline materials. Two approaches have been employed to acquire the orientation information at each sample position: (i) diffraction patterns (DPs) are recorded directly in diffraction mode during beam scanning (Fundenberger *et al.*, 2003 ▸; Morawiec *et al.*, 2014 ▸; Kim *et al.*, 2015 ▸; Rauch *et al.*, 2010 ▸); and (ii) DPs are reconstructed from conical-scanning dark-field images recorded at various tilt angles (Dingley, 2006 ▸; Wu & Zaefferer, 2009 ▸). For the latter approach, the automatic indexing of DPs from highly strained samples remains a challenge (Dingley, 2006 ▸). A three-dimensional orientation mapping technique called 3D-OMiTEM was developed based on the conical-scanning dark-field imaging technique (Liu *et al.*, 2011 ▸). More recently, Midgley’s group at Cambridge (Eggeman *et al.*, 2015 ▸) determined the three-dimensional precipitate morphology in an Ni-based superalloy using scanning precession electron diffraction (Vincent & Midgley, 1994 ▸). The three-dimensional reconstruction was carried out using a principle-component based separation algorithm to separate the matrix and precipitate DPs.

Transmission electron diffraction (TED) is an appropriate technique for complex nanostructure analysis because it is highly sensitive to local structure and can be obtained using a small electron beam (Cowley, 1993 ▸; Spence & Zuo, 1992 ▸; Zuo *et al.*, 2003 ▸, 2004 ▸; Midgley & Eggeman, 2015 ▸; Zuo & Tao, 2010 ▸). Compared with the 3D-EBSD and XRD-based techniques, the small interaction volume in TED allows for a higher spatial resolution. Traditionally, TED is performed either by using parallel-beam illumination with the help of a selected-area aperture for selected-area electron diffraction (SAED) or by using a focused beam for convergent-beam electron diffraction (CBED). Electron nanodiffraction (END) can be performed in a modern TEM using an electron beam of a few nanometres in diameter with the help of a minilens (Zuo *et al.*, 2003 ▸, 2004 ▸). For nanostructure analysis, it is extremely helpful to record multiple DPs by scanning the beam over the sample areas of interest. Previously, we have developed a TEM-based SEND technique that uses the built-in TEM deflection coils to shift the beam (Kim *et al.*, 2015 ▸; Zuo & Tao, 2010 ▸). In a conventional TEM with an LaB_6_ source, SEND can be performed in the low-dose mode using electron beams of ∼2–5 nm in full-width at half-maximum (FWHM) and 0.1 pA or less in beam current.

Here, we report a new technique called three-dimensional scanning electron nanodiffraction (3D-SEND) for three-dimensional nanostructure determination. This technique aims to determine the three-dimensional morphologies and orientations of nanograins. It is a diffraction-based technique, taking advantage of the non-destructive, high-resolution and sensitive nature of TED. We have further developed this technique for tomography with lower-dose diffraction and an improved DP indexing scheme. The general methodology is explained in detail in Sections 2 and 3. We apply our technique to the characterization of a nanocrystalline TiN thin-film sample.

## 2. Experimental methods   

### 2.1. The holder design and sample preparation   

In electron tomography, the range of sample rotation (or tilt) plays a crucial role in the accurate reconstruction of the targeted objects. The tilt angle of a TEM specimen can be limited by the sample thickness, by the shadowing effect from the sample, or by the sample holder or the supporting grid (Midgley & Dunin-Borkowski, 2009 ▸). For three-dimensional electron diffraction, we have designed a custom tomography holder that allows ±87° rotation of the specimen, based on the holder previously described by Mao *et al.* (2015 ▸). The design employs a needle-shaped specimen mounted on a regular JEOL single-tilt holder (JEOL, Tokyo, Japan).

**Figure 1:**
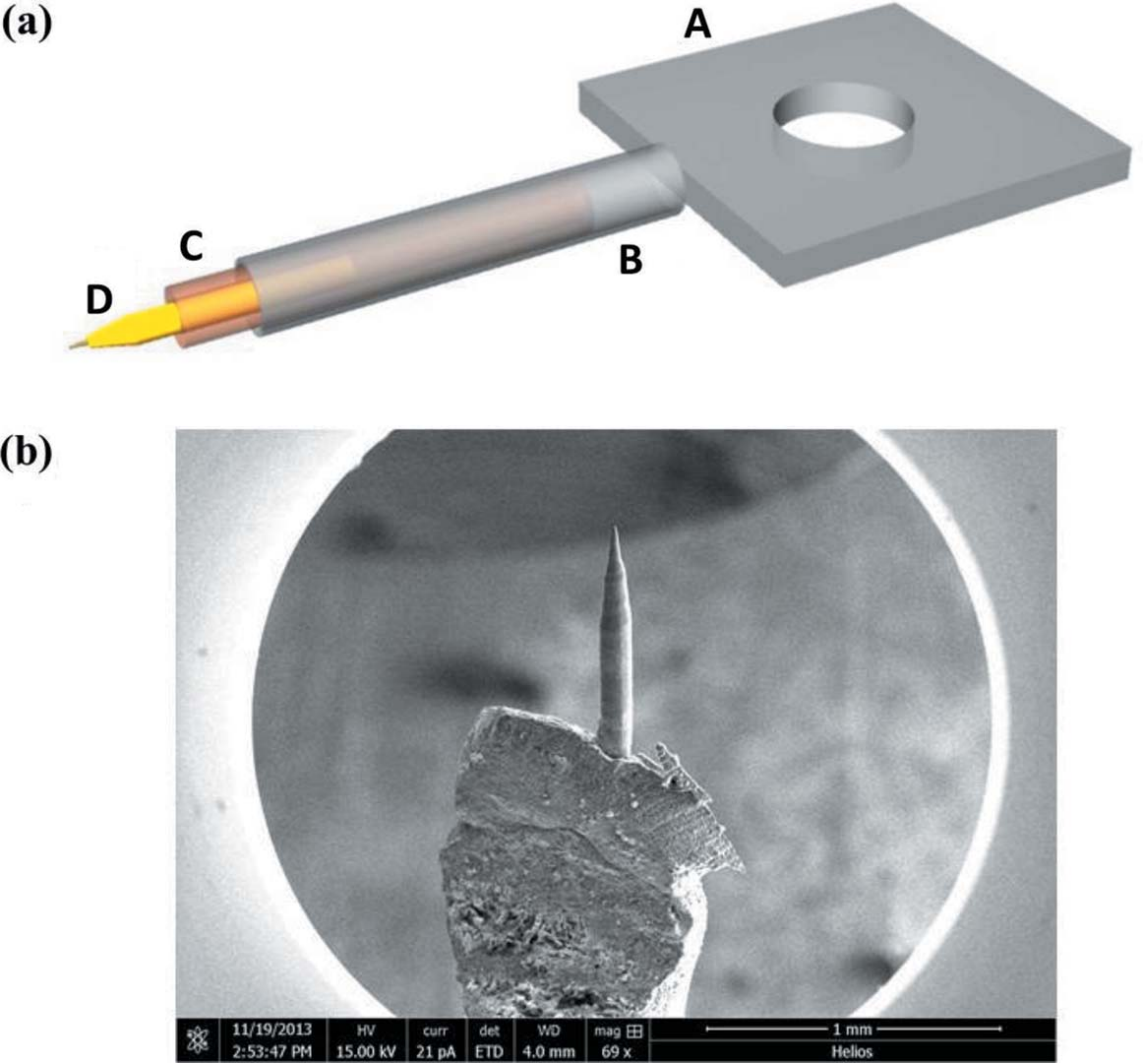
The tomographic holder for tip-shaped samples. (*a*) Schematic design of the customized tomographic holder. Components A to D are described in the text. (*b*) SEM image of the polished W wire.

Specifically, we replace the tip of a regular single-tilt holder with a custom stainless steel mount (component A in Fig. 1 ▸ *a*). Component B is a stainless steel tube welded to A. The consumable parts are C and D. Component C is a copper tube with an outside diameter smaller than the inside diameter of B. We install the copper tube by simply sliding it into B. The tail of the copper tube is clamped slightly into an oval shape. In this way there is a friction force to hold the copper tube steady within B. A tungsten (W) wire (component D) is clamped onto the other end of the copper tube. The W wire is further electrochemically polished in 5 wt% NaOH solution at 2.5 V for 90 s. A sharp tip is formed near the top of the W tire after polishing (Fig. 1 ▸ *b*). We milled this tip away using a focused ion beam (FIB), leaving a flat plateau with a diameter of 10 µm. The W wire serves as a substrate for the sample.

The sample is placed on top of the W wire support using the FIB lift-out technique. The sample is annularly milled to the desired diameter (usually between 100 and 300 nm). The small diameter of the mounting tube (component C) allows free sample rotation in the smallest polepiece gap, for example the JEOL ultra-high resolution polepiece. The needle-shaped sample is also nearly parallel to the rotation axis of the holder, which provides rotation with a minimum precession movement. However, the sample may not be eucentric; we have observed height movements within a 50 µm range for ±87° sample rotation.

### 2.2. Data acquisition   

**Figure 2:**
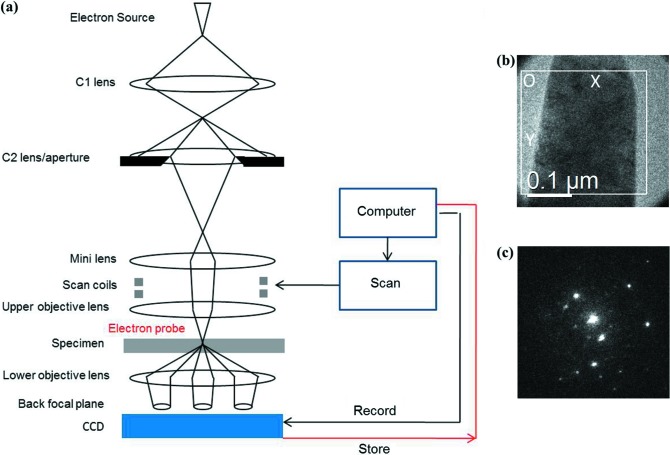
Data acquisition in 3D-SEND. (*a*) Schematic illustration of the SEND system. (*b*) TEM image of a needle-shaped sample. The white rectangle indicates the area covered by the beam scanning, which starts from the origin position O. (*c*) An experimental DP acquired during beam scanning.

For SEND data acquisition, the TEM is aligned based on the procedures described in our previous paper (Kim *et al.*, 2015 ▸). Improper TEM alignments can lead to DP shift and beam tilting during beam scanning, which may complicate the post-acquisition data analysis. Thus, care must be taken to separate the beam shift from the beam tilt in the illumination deflection coils. This is followed by aligning the intermediate lens focus (diffraction focus) which also affects the DP shift. DP recording and beam scanning are automated using a *DigitalMicrograph* script (Gatan Inc., Pleasanton, California, USA) to control the illumination deflection coils and camera readout (see Fig. 2 ▸ *a*) (Tao *et al.*, 2009 ▸). The beam is shifted in a step-by-step manner from left to right and top to bottom. Fig. 2 ▸(*b*) shows an example of a scan over the needle-shaped TiN sample. For DP recording, we use a typical exposure time of 0.1 s and a DP size of 256 × 256 pixels, which were found sufficient for further indexing. A small camera length is used to include as many diffraction spots as possible without too much degradation in DP resolution. Fig. 2 ▸(*c*) shows an example of an experimental DP. The spot size, beam-shift step size and scanning area are subject to the specific study requirements.

Ideally, the sample tilt can vary from −90° to 90°. However, in practice this range is limited by the goniometer design and controller. In our case, on a JEOL 2100 TEM, the *z* height movement is coupled with *x* and *y* movement at high tilt angles. Thus, it is extremely hard to control the sample position from 87° to 90°. The step size of the sample tilt is selected based on a balance between the time cost of the data acquisition, the data size and the accuracy of the reconstructed grain morphology. A smaller step size gives a more reliable three-dimensional morphology of a grain, at the cost of increased time and data size. Based on our experience with this technique, it is preferable to use a tilting step size smaller than 10°. The sample position was manually adjusted after sample rotation. In future, automatic correction for the sample position will significantly reduce the experimental time. However, this requires improvements in holder technology as well as in the electron-beam scanning algorithm.

## 3. Data analysis and three-dimensional reconstruction   

### 3.1. Two-dimensional grain morphology identification   

**Figure 3:**
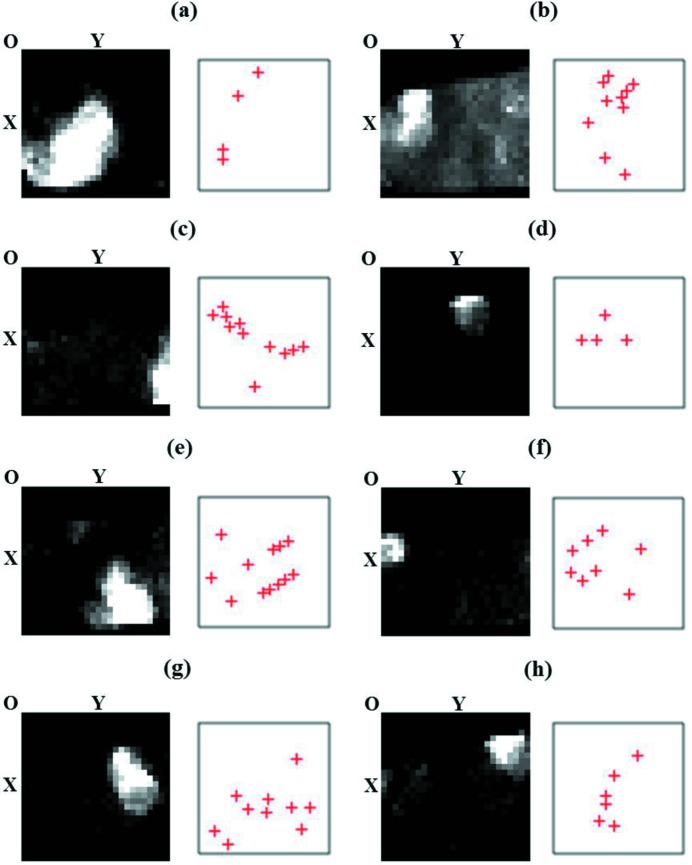
(*a*)–(*h*) Eight grains identified for the TiN sample at −5° rotation. In each subgraph, the left-hand image is the averaged dark-field image representing the grain shape, while the right-hand pattern with crosses (+) represents the corresponding image-filtered DP. Each cross marks a diffraction spot. The spot intensities are not shown here.

The two-dimensional morphology of a grain is identified by constructing dark-field images from the recorded DPs through the following sorting process. At a specific sample rotation angle, the position and intensity of every diffraction spot are recorded using the template matching method (Zuo *et al.*, 2014 ▸). Using this information, a dark-field image is then constructed for each diffraction spot. Two dark-field images will be similar to each other if their diffraction spots belong to the same DP of the crystal grain. Thus, dark-field images with similar contrasts can be grouped by using image cross-correlation. The similarity between two images is defined by the normalized cross-correlation factor 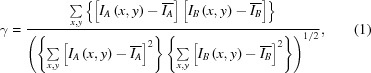
where *I_A_*(*x*, *y*) and *I_B_*(*x*, *y*) are the intensities of a pixel (*x*, *y*) in image *A* or *B*, respectively, and 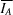 and 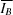 are the mean intensities of image *A* or *B*, respectively (Lewis, 1995 ▸). A correlation threshold is used for the grouping based on the trial and error method. The two-dimensional morphology is extracted from the averaged dark-field image, after applying an intensity threshold. By using the normalized correlation factor γ, the analysis does not depend on the ‘dark-field image’ intensity and thus provides information on the grain shape. Meanwhile, diffraction spots belonging to a single grain are grouped into a single-crystal DP. Unlike an experimental DP, this DP only contains a subset of the measured diffraction spots, therefore we call it the ‘image-filtered’ DP. Fig. 3 ▸ shows the sorting results for the TiN sample at −5°. This step is repeated for every sample rotation angle.

### 3.2. DP indexing   

The filtered DPs acquired in Section 3.1 are indexed for the determination of crystal orientation. If the number of diffraction spots in an image-filtered DP is not sufficient for reliable indexing, we index the averaged experimental DPs within the identified two-dimensional grain. In this case, the averaged experimental DP may contain diffraction spots from other overlapping grains. Therefore, multiple local maxima may appear in the indexing correlation factor map (Zaefferer, 2011 ▸). Electron DP indexing is done through a *DigitalMicrograph* script package called *DPIndex* that we have developed at the University of Illinois. This package uses both length and angle information on the diffraction spots, as well as the scaled diffraction intensity, for DP indexing. The experimental DP was correlated with the DPs simulated by the *QED* program (Zuo, 2006 ▸) to produce an indexing correlation factor map, where the best match is identified based on the identified peaks. The kinematic calculation is used for the DP simulation. This indexing scheme differs from the template-matching technique of Zaefferer’s methods in the following ways (Zaefferer, 2000 ▸; Wu & Zaefferer, 2009 ▸). Each diffraction spot in a DP is located and measured by matching with a spot template. We record both the position and intensity of a found diffraction spot. Two intensity profiles are calculated to represent each DP. First, the spot intensities are projected onto the radial distance from the centre. Second, the angular distribution of diffraction spots at a certain radial distance is generated. During an indexing process we sort the simulated DPs based on the radial and angular intensity profiles. Using this approach, the time efficiency of DP indexing is dramatically improved. The final match is made by a cross-correlation between the experimental and simulated DPs at the top of the sorted list. Most DPs can be indexed this way, with the exception of a few DPs far away from the zone axes. However, a small proportion of failed indexing does not affect the overall analysis.

### 3.3. Three-dimensional reconstruction   

#### 3.3.1. Reconstruction algorithms and parameters   

**Figure 4:**
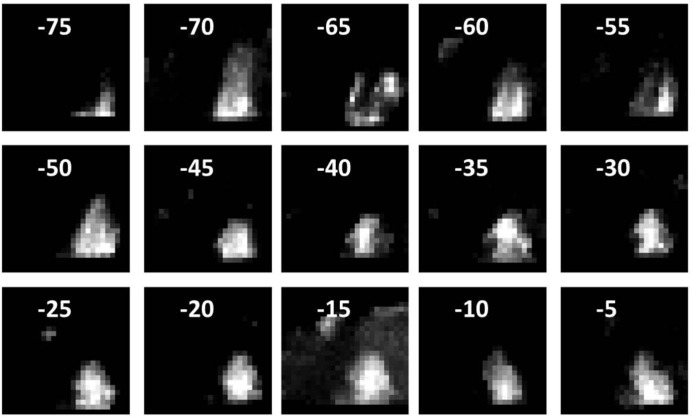
Dark-field images identified as belonging to the same grain, at tilt angles from −75° to −5°.

To reconstruct a three-dimensional grain from the projected dark-field images, we use the algebraic reconstruction technique (ART; Herman, 2009 ▸). The commonly used back-projection method is not applicable here, since the dark-field images are not monotonic to any physical properties of the grain because of electron multiple scattering. Additionally, the use of ART is justified by the following reasons. First, one grain may only be identified from a part of the rotation data set. It is also possible that the data at a particular rotation angle are not usable because of weak diffraction spots or strong multiple scattering. In either case, we found that the number of available projections is often limited in the three-dimensional diffraction data set. ART is designed for incomplete projection data. Secondly, ART allows inputs of prior information about the object. The outline of the needle-shaped sample introduces a strong constraint that can be included in the reconstruction (it is used for setting up the ray–voxel interaction matrix, details of which are described later). This step improves the accuracy of the reconstruction results. Prior to three-dimensional reconstruction using ART, the two-dimensional dark-field images are identified as belonging to the same grain. This is done by confirming two projected grain images belonging to the same grain from neighbouring rotations using two criteria: (i) the difference between the two beam directions is equal to the sample rotation step size; and (ii) the two-dimensional grain images overlap with each other. Fig. 4 ▸ shows the two-dimensional images of one grain from −75 to −5°.

The dark-field image contrast is affected by diffraction from overlapping grains along the beam direction, the grain position relative to the top sample surface and the grain size along the beam direction. A quantitative analysis of pixel intensities in dark-field images is difficult at the current stage. Thus, we outline the grain shape by setting a threshold to the dark-field image, which ignores the intensity variation within the grain. However, at some rotation angles the extracted shape only gives part of the grain. For example, the −75° image in Fig. 4 ▸ shows an incomplete grain shape. Also, it is possible that two or more grains share similar orientations at a certain angle. For example, the −15° image in Fig. 4 ▸ shows two grains. In this case, we manually exclude the accidental grain near the top left-hand corner.

ART requires discretization of the sample and the projection. The projection is already discretized by the stepwise beam scanning. We assume that the beam scanning is performed in an *m* × *n* area and that the number of sample tilt angles is *r*. Then the sample is discretized into *m* × *n* × *n* voxels. Each voxel is a cube with an edge length equal to the scanning step size. The problem of the three-dimensional reconstruction can be reduced into a linear algebraic equation 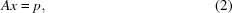
where *A* is a ray–voxel interaction matrix (RVM), *x* is a column vector representing the object distribution and *p* is a column vector representing the projection data. The height of *A* is equal to the number of rays (electron beams) applied in the experiment. The width of *A* is equal to the number of voxels in the sample space. Under the previous assumption, the size of *A* is *mnr* × *mn* ^2^. The value of an element *a_ij_* in *A* is the same as the length of the segment of the *i*-th ray inside the *j*-th voxel. *a_ij_* represents the contribution of the *j*-th voxel to the projection result of the *i*-th ray. *A* is pre-calculated using a fast ray-tracing algorithm proposed by Amanatides & Woo (1987 ▸). An element *x_j_* in *x* represents the distribution of the object in the *j*-th voxel. *x* is the unknown variable. An element *p_i_* in *p* represents the measured projection under the *i*-th ray. *p* is determined based on the dark-field images acquired in Section 3.1. The value of *p_i_* is set to 1 if the projection of the *i*-th ray is within the outline of the two-dimensional grain morphology, otherwise it is set to 0. Various algorithms were developed for solving equation (2). Here, we use the algebraic iterative algorithm first proposed by Kaczmarz (1937 ▸). *x* is additively modified in each cycle to approximate the ideal solution. We stop the iteration when *x* is stable.

The output of the ART is a three-dimensional map of voxel contribution to the target grain. By creating an isosurface of the map, the morphology of the grain can be plotted in three-dimensional space. The isosurface value is adjusted so that the isosurface is continuous for a given grain.

#### 3.3.2. Prior information   

Prior information is especially important for an accurate ART reconstruction from incomplete projection data (Herman, 2009 ▸). Since the scattering intensity drops along the beam direction, we employ an exponential damping effect for *a_ij_* along the ray when calculating the RVM. This treatment is necessary, since the observed DP is often dominated by one set of lattices when the beam travels through multiple grains from top to bottom. For example, we may not be able to identify the bottom grain from the dark-field images. Assigning a smaller contribution to voxels near the bottom is closer to the experimental situation than assigning a constant contribution along the incident ray. The exponential damping coefficient is chosen so that two adjacent grains do not overlap with each other.

The position and shape of a grain are bounded by the outline of the sample. Therefore, a three-dimensional mask is applied to exclude any voxel contribution from the vacuum. For our needle-shaped sample, the three-dimensional mask is approximated as a truncated cone. The size of the truncated cone is determined through the bright-field TEM images recorded during the experiment.

#### 3.3.3. Projection alignment   

Ideally, the scanning area is centred on the same point. In practice, however, the scanning area may deviate slightly from the desired position, since we adjust the sample height for each rotation. Thus, we must align the projection images of a grain before the tomographic reconstruction. The horizontal position (*x*) of a projection is aligned by centring the pillar’s silhouette. For the vertical position (*y*) we match a projection with its neighbour projection. The matching process is done by cross-correlation, since the projected shape of one grain changes slightly after a small rotation. The *y* alignment is performed sequentially over the rotation range.

### 3.4. Grain orientation determination   

The orientation of a grain is determined from the indexing results of all available projections. In the stereoprojection, the indexing results are expected to form a line. The orientation of a grain is defined by the transformation matrix, which transforms a vector in the crystal coordinates onto the holder coordinates. We define the transformation relation as *h* = *Tc*, where *c* is a 3 × 1 vector which represents a direction in the crystal coordinates and *h* is a 3 × 1 vector which represents the same direction in the holder coordinates. *T* is a 3 × 3 matrix which transforms the direction from the crystal coordinates into the holder coordinates.

Ideally, *T* can be determined from two arbitrary observations. In practice, the indexing results contain noise and a more accurate *T* is obtained by minimizing the following error function 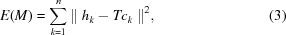
where *n* is the number of useful projections. The solution for *T* can be found using a single-value decomposition method (Markley, 1988 ▸). First, we calculate a 3 × 3 matrix *B* as 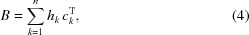
where 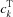 is the transpose of *c_k_*. Next, we compute the single-value decomposition of *B* as follows 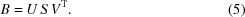
The transformation matrix is 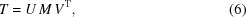
where 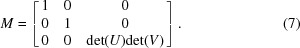
The matrices of *U*, *S* and *V* in equation (5) are all calculated results of the single-value decomposition on the matrix *B*.

## 4. Results   

The 3D-SEND experiment was performed on a TiN thin-film nanocrystalline sample. TiN is widely used in the electronics industry, as well as in protective and decorative coatings (Fortuna *et al.*, 2000 ▸). Compared with polycrystalline TiN, nanocrystalline TiN exhibits improved mechanical properties such as hardness, wear and corrosion resistance (Pan *et al.*, 1998 ▸; Wang *et al.*, 2014 ▸; Ma *et al.*, 2006 ▸). This is achieved by controlling the intrinsic properties such as grain size, morphology and texture (Mayrhofer *et al.*, 2003 ▸). Nano­crystalline TiN thin films can be grown by CVD (chemical vapour deposition) and PVD (physical vapour deposition) (Mayrhofer *et al.*, 2002 ▸). Here, the TiN sample was grown on a *p*-type Si(100) substrate in an unbalanced magnetron sputtering (UBMS) system (Wang *et al.*, 2014 ▸). The deposition temperature was 400°C. Prior to the deposition, the substrate surface was pre-sputtered by Ar ions to remove the surface oxide layer. After pre-sputtering, the working gas was introduced into the chamber, consisting of Ar and N_2_ (99.9995% purity) with flow rates of 30 and 1 standard cm^3^ min^−1^, respectively. The working pressure was maintained at 1.7 × 10^−1^ Pa (1.3 × 10^−3^ Torr). Substrate bias voltages of −60 and −70 V were applied to adjust the residual stress of the specimens. The thickness of the deposited TiN thin film was around 4.5 µm.

The FIB cut and lift-out was performed perpendicular to the growth direction. The sample was annularly milled using a 30 kV ion beam to ∼200 nm. It was further polished by 5 and 2 kV ion beams in order to reduce the surface amorphous layer thickness. 3D-SEND was performed on a JEOL 2100 (Cryo) TEM at 200 kV in the NBD mode. The beam size was set to 7 nm in FWHM. The scanning covered an area of 26 × 26 pixels and each step was 11 nm. For recording electron DPs, we used a Gatan CCD camera designed for a 200 kV electron source. The camera has a 4 megapixel (2 k × 2 k) Peltier-cooled CCD chip and it was mounted on-axis under the microscope column. For our SEND experiment, we used 8× for binning (256 × 256 pixels). The exposure time was set at 0.1 s. The sample was tilted over a range of ±85° at a step size of 5°. In total, 23660 DPs were recorded.

**Figure 5:**
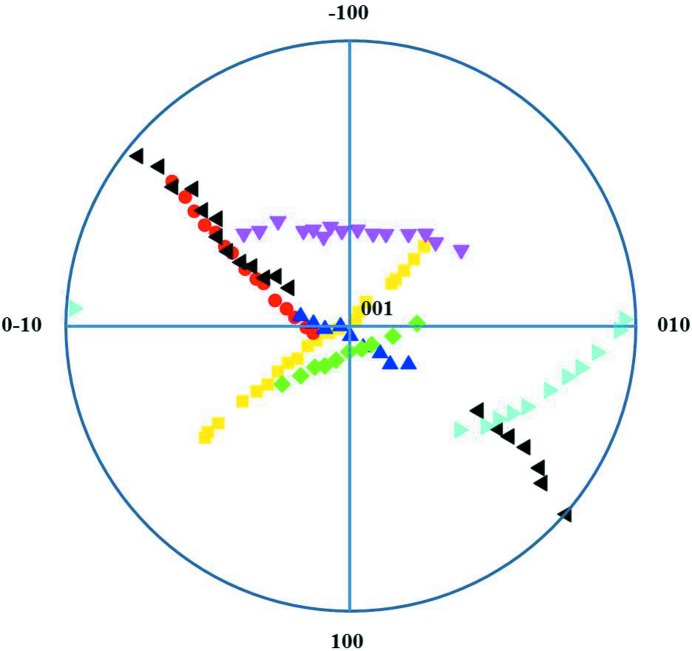
The beam directions of seven grains in the TiN sample, plotted in a stereographic projection. The gaps due to a few missing points are due to the failed automatic indexing.

**Figure 6:**
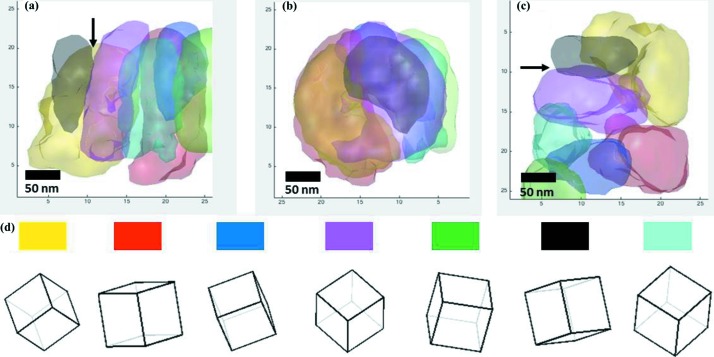
Reconstructed grains and their orientations. (*a*) Side view, (*b*) front view and (*c*) top view of the three-dimensional morphologies of the reconstructed grains (a Σ9 grain is indicated by the arrows). (*d*) The orientations of the seven grains. Each cube is labelled by the colour used to represent the grain in parts (*a*)–(*c*).

The acquired data were processed based on the methods in Section 3. We identified seven major grains in the sample. Beam directions at different sample tilt angles are shown in the pole figure (Fig. 5 ▸) for all seven grains. The ART reconstruction takes about 20 iterations to finish. The three-dimensional morphologies and orientations of the seven grains are illustrated in Fig. 6 ▸. Previous studies found that the grains are elongated along the growth direction and that multiple grains are stacked along the elongation direction (Fortuna *et al.*, 2000 ▸). Our reconstruction results show that the grains are elongated and the grain boundaries are not ideally flat.

## 5. Discussion   

### 5.1. Spatial resolution   

The spatial resolution of the reconstructed data ideally equals the step size used during beam scanning under certain conditions. How this works is that the reconstructed data are a set of scattered points assigned with values. These points are then interpolated to obtain the grain morphology using a cubic kernel, which usually gives a rendering resolution lower than the data resolution.

The spatial resolution of 3D-SEND is ultimately limited by the electron probe size *d*_0_ and the column diameter under the column approximation. The diameter of the cone *d_AB_* is defined approximately as
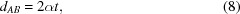
where α is the convergence semi-angle and *t* is the sample thickness. The radius of the first Fresnel zone ρ_1_ used to represent the diffraction column is calculated using
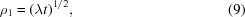
where λ is the electron beam wavelength. At 200 kV, λ = 2.5 pm. For a JEOL 2100 TEM, we can form a probe with a FWHM of 2.3 nm using a 10 µm condenser aperture in CBD mode with a full convergence angle of 4.2 mrad. If the sample thickness is 200 nm, *d_AB_* is 0.8 nm and ρ_1_ is 0.7 nm. This means that the best spatial resolution of 3D-SEND is probe-limited to around 2 nm in a JEOL 2100 TEM.

The influence of scan distortion also limits the spatial resolution of the reconstruction. The beam scan is calibrated using the three-point method described in our previous paper (Kim *et al.*, 2015 ▸). The actual beam position could be off by 1–3 nm after moving the beam by 200 nm. Additional scan distortion is introduced by sample drifts during the beam scan. Our experiment shows that the drift is 3 nm or less in both horizontal and vertical directions during the period of a 10 min scan (Kim *et al.*, 2015 ▸). Considering these factors, we estimated that the calibration error and sample drift put a limit on the spatial resolution of around 1.5 nm when the grain size is around 100 nm. This limit is smaller than the diffraction limit (∼2 nm) discussed above. Therefore, the final spatial resolution is limited to around 2 nm in a JEOL 2100 TEM.

### 5.2. Grain boundary identification   

A grain boundary can be identified using five independent parameters. Three are used to describe the orientation of a grain, and the other two are used to describe the orientation of the grain boundary surface. The grain boundary character distribution (GBCD) controls properties such as grain energy, wear resistance, segregation and mobility (Raabe *et al.*, 2014 ▸; Randle *et al.*, 2008 ▸; Olmsted *et al.*, 2009 ▸). While the mis­orientation across a grain boundary can be readily acquired from an EBSD study, the determination of the grain boundary plane orientation is still an ongoing research subject (Darbal *et al.*, 2013 ▸; Herbig *et al.*, 2014 ▸). 3D-SEND directly provides the five parameters of a grain boundary if two adjacent grains are both reconstructed. The grain boundary can be segmented into two-dimensional planes based on the three-dimensional resolution. The accuracy of the plane segment orientations is determined by the resolution of the reconstructed grains. For example, the boundary between the magenta and black grains of Fig. 6 ▸ is identified as a Σ9 grain boundary (38.9°/〈110〉), highlighted by arrows in Fig. 6 ▸. The measured surface area of this grain boundary is around 800 nm^2^ if we approximate the boundary as a plane.

### 5.3. Comparison with existing three-dimensional nanostructure analysis techniques   

The best spatial resolution that has been reported for XRD-based tomographic mapping techniques is submicron using DAXM (Larson *et al.*, 2002 ▸). Tomography based on coherent X-ray diffraction (Pfeifer *et al.*, 2006 ▸; Robinson & Harder, 2009 ▸) can in principle provide higher spatial resolution at tens of nanometres, but such a technique has yet to be developed for nanocrystalline materials. Using electrons, the spatial resolution can be improved to ∼1–2 nm. The method (scanning precession electron tomography, SPET) developed by Eggeman *et al.* (2015 ▸) reduces the electron multiple scattering effect by using precession electron diffraction. By increasing the number of DP spots, this method improves the reliability of orientation mapping. The drawback of SPET is the increased acquisition time and also the electron beam size. In comparison, our SEND technique without precession works under the low-dose regime. Individual DPs are acquired using a 0.1 s exposure time, but the DP indexing is challenging. To overcome this challenge, we have developed a dark-field image sorting algorithm, which works for un­processed electron diffraction data.

In principle, 3D-OMiTEM (Liu *et al.*, 2011 ▸) can be applied to single-phase materials such as the nanocrystalline TiN sample. The recorded dark-field image has a large field of view and diffraction-limited resolution, but both the resolution and the angular range of the reconstructed DPs are limited by the beam tilt angles. A large electron dose is also required, since each dark-field image is recorded using only one diffraction spot. 3D-SEND is dose efficient, since all diffraction spots are required and only the sample under the electron beam is exposed. In addition, 3D-SEND has a better DP resolution than 3D-OMiTEM, allowing a more reliable separation of overlapping grains.

By using a needle-shaped sample, we are able to record DPs from the sample between −85° and 85°. The large sample rotation range increases the accuracy of the grain morphology reconstruction. In addition, the needle-shaped sample provides a sample boundary constraint for the three-dimensional reconstruction of grains. Most TEM samples are prepared in the thin-film geometry, but the main drawback of using this geometry for diffraction tomography is that the number of grains under the beam does not stay constant as the sample rotates for a fixed scan area. We also expect the needle-shaped samples to work with EDX and atom-probe tomography, which will bring the added benefits of combined crystallography and composition analysis.

## 6. Conclusions   

We have introduced 3D-SEND as a versatile and reliable three-dimensional orientation mapping technique. Both three-dimensional morphology and orientation can be determined for nanograins. The technique uses high-resolution electron diffraction which is adaptive to various nanocrystalline samples. With the help of the holder design, this technique can easily be coupled to other characterization techniques such as atom-probe tomography and nanoindentation. Future applications of this technique are anticipated for studying single- and multiphase grain boundaries at the nanoscale.
